# Inhibition of Soybean 15-Lipoxygenase and Human 5-Lipoxygenase by Extracts of Leaves, Stem Bark, Phenols and Catechols Isolated From *Lithraea caustica* (Anacardiaceae)

**DOI:** 10.3389/fphar.2020.594257

**Published:** 2020-11-30

**Authors:** Alejandra Muñoz-Ramírez, Carolina Mascayano-Collado, Andrés Barriga, Javier Echeverría, Alejandro Urzúa

**Affiliations:** ^1^ Departamento de Ciencias del Ambiente, Facultad de Química y Biología, Universidad de Santiago, Santiago, Chile; ^2^ Unidad de Espectrometría de Masas-CEPEDEQ Facultad de Ciencias Químicas y Farmacéuticas, Universidad de Chile, Santiago, Chile

**Keywords:** *Lithraea caustica*, anacardiaceae, 3-n-alk(en)yl-catechols, soybean 15-lipoxygenase, human 5-lipoxygenase, anti-inflammatory activity

## Abstract

*Lithraea caustica* (Molina) Hook. and Arn. (Anacardiaceae), common name Litre, is an evergreen endemic plant used in the Mapuche Chilean folk medicine. The stem juice of *L. caustica* mixed with *Rubus ulmifolius* (blackberry) is used to treat cough and the infusion of leaves is used in baths to treat joint inflammations. In this study, the activities of 3-n-alk(en)yl-catechols, obtained from the dichloromethane extract of the epicuticular compounds of fresh leaves (DCME), stem bark petroleum ether extract (PEE), fractions of phenols and phenol-acid compounds obtained from the methanolic extract (methanolic extract) of defatted leaves and aqueous infusion (AE) from fresh leaves, were evaluated as *in vitro* inhibitors of soybean 15-lipoxygenase (15-sLOX) and human 5-lipoxygenase (5-hLOX), one of the inflammation pathways. The 3-n-alk(en)yl-catechols were characterized by gas chromatography-mass spectrometry and 1D and 2D nuclear magnetic resonance analysis as mixtures of 3-[(10E)-pentadec-10′-en-1-yl]-catechol, 3-[(10Z)-pentadec-10′-en-1-yl]-catechol and 3-n-pentadecylcatechol. In addition, two fractions, obtained from MeOHE, were characterized by liquid chromatography electrospray ionization tandem mass spectrometric as complex mixtures of known acids and phenolic compounds. DCME, MeOHE and ethyl acetate extract (AcOEtE) extracts showed inhibition against 15-sLOX, and the AE of fresh leaves, showed the best inhibition against 5-hLOX. The mixture of 3-n-alk(en)yl-catechols showed inhibition of 15-sLOX and 5-hLOX. The compounds 3-[(10Z)-pentadec-10′-en-1-yl]-catechol (IC_50_ 2.09 µM) and 3-n-pentadecylcatechol (IC_50_ 2.74 µM) showed inhibition against 5-hLOX. The inhibition values obtained for the 3-n-alk(en)yl-catechols are in the range of well-known inhibitors of 5-hLOX. Acetylation of the 3-n-alk(en)yl-catechols blocks the inhibitory activity, indicating that the free catechol function is necessary for the enzyme inhibition. In addition, the fractions of phenols and phenol-acid compounds showed inhibitory activity against 15-sLOX and the AE, showed a good inhibition against 5-hLOX. These results would be in agreement with the use of *L. caustica*, as an anti-inflammatory in Mapuche ethnomedicine.

## GRAPHICAL ABSTRACT

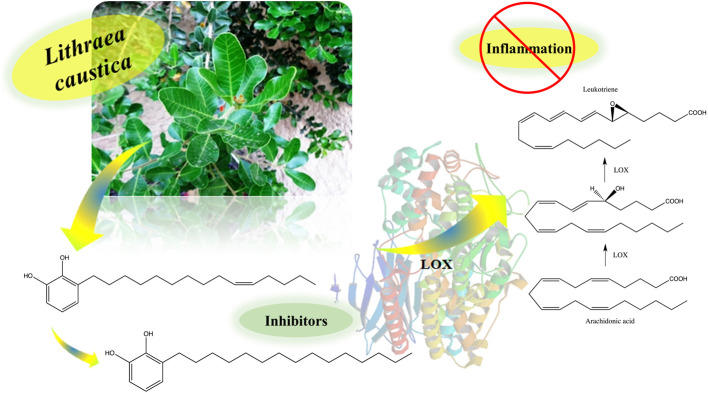

## Introduction

*Lithraea* (Anacardiaceae), is represented in South America by three species *L. brasiliensis* (L.) Marchand (Argentina, Brazil and Uruguay), *L. caustica* (Mol.) Hook. and Arn., (Chile) and *L. molleoides* (Vell.) Engl. (Argentina, Brazil, Paraguay and Uruguay), (Zuloaga et al., 2008). These three species are characterized by producing allergic contact dermatitis to sensitive people, associated with the presence of 3-*n*-alk(en)yl-catechols (Gambaro et al., 1986; Alé et al., 1997). *L. caustica*, vernacular name Litre, is a common evergreen tree, shrub or creeping habit plant, from 0.5 to 4 m high, endemic to Chile. It is distributed between Atacama and Los Rios Regions (Rodriguez et al., 2018). Extracts obtained from leaves and stems are used in Mapuche folk medicine in the treatment of joint inflammatory diseases (Montecino and Conejeros, 1985), a tincture of leaves is used at low doses to treat scaly skin lesions (Muñoz et al., 1981) and the leaves are eaten raw to treat allergy problems. Additionally, a mixture of stem juice of *L. caustica* and *Rubus ulmifolius* Schott (Rosaceae, common name: blackberry) is used for cough treatment (San Martín, 1983). Although the preparations of *L. caustica* are used for inflammatory diseases in Mapuche ethnomedicine, the phytochemical and pharmacological studies that have been conducted on the species are scarce. From the stem bark, 3-[(10*Z*)-pentadec-10′-en-1-yl]-catechol **(2)** was identified as a responsible compound for the Litre’s allergenic properties (Gambaro et al., 1986; López et al., 1998). Also, some common terpenoids as myrcene, *α*-pinene, *p*-cymene and limonene and caryophyllene were identified in dry leaves by solid phase micro-extraction (Garbarino et al., 2002) and from the fresh leaves cuticular extract a mixture of monoterpenes, sesquiterpenes, hydrocarbons and 3-[(10*Z*)-pentadec-10’-en-1-yl]-catechol **(2)**, were characterized (Urzúa et al., 2011).

To validate, with scientific evidence, the anti-inflammatory use of *L. caustica* in Mapuche ethnomedicine, a phytochemical study of the leaves and stem bark of *L. caustica* was carried out. The different extracts, mixture of compounds and pure compounds were analyzed as inhibitors of 5 human lipoxygenase (5-hLOX) and 15 soybean lipoxygenase (15-sLOX). 15-sLOX and 5-hLOX, are enzymes that use molecular oxygen in the dioxygenation of arachidonic acid (AA) to form hydroperoxides (Boyington et al., 1993; Saura and Jean-Didier, 2016; Snodgrass and Brüne, 2019) from 1,4-diene units (Chohany et al., 2011) and which are related in the biosynthesis of lipoxins (LXs) and leukotrienes (LTs) (Vásquez-Martínez et al., 2019). They play a role in the pathogenesis of inflammatory, hyperproliferative, neurological, and metabolic diseases (Dobrian et al., 2011). It is important to mention, that 15-sLOX is used as model of 5-hLOX due to their structural similarity and mechanism of action (Wecksler et al., 2009).

In this communication we described the characterization by gas chromatography-mass spectrometry (GC-MS) of fractions obtained from the dichloromethane extract (DCME) and petroleum ether extract (PEE) of aerial parts (leaves and stem bark) of *L. caustica*. Three 3-*n*-alk(en)yl-catechols*,* 3-[(10*E*)-pentadec-10′-en-1-yl]-catechol (**1**), 3-[(10*Z*)-pentadec-10v-en-1-yl]-catechol (**2**) and 3-*n*-pentadecylcatechol (**3**) were isolated and identified using spectroscopic and spectrometric methods. In addition, two fractions of compounds (AcOEt-1 and AcOEt-2), obtained after processing a methanol extract of defatted leaves of *L. caustica* were characterized by liquid chromatography electrospray ionization tandem mass spectrometric (LC-ESI-MS/MS) as complex mixtures of phenolic and phenolic acid compounds. The mixture of 3-*n*-alk(en)yl-catechols and particularly the isolated compounds showed strong inhibitory activity against of 15-sLOX and 5-hLOX. In addition, the mixtures of phenolic and phenolic acid compounds, showed mild activity as 15-sLOX inhibitors.

## Materials and Methods

### Plant Material

Representative samples of leaves and stem bark of *Lithraea caustica* (Mol) Hooker and Arn., were collected during the flowering season, November 2015, from a population growing in Farellones (Región Metropolitana) Santiago, Chile (33° 18′ 35.9″S; 70° 19′ 19.9″W) at altitudes of 1,200–1,300 m above sea level (masl). Voucher specimen (SGO 226473) was deposited in the Herbarium of the National Natural History Museum, Santiago, Chile.

### Preparation of Crude Extracts, Fractionation, Isolation and Characterization of Components

Extracts were obtained following the methodology described by Huanquilef et al., (2020) with some modifications.

#### Petroleum Ether Extract From Stem Bark

**FIGURE 1:**
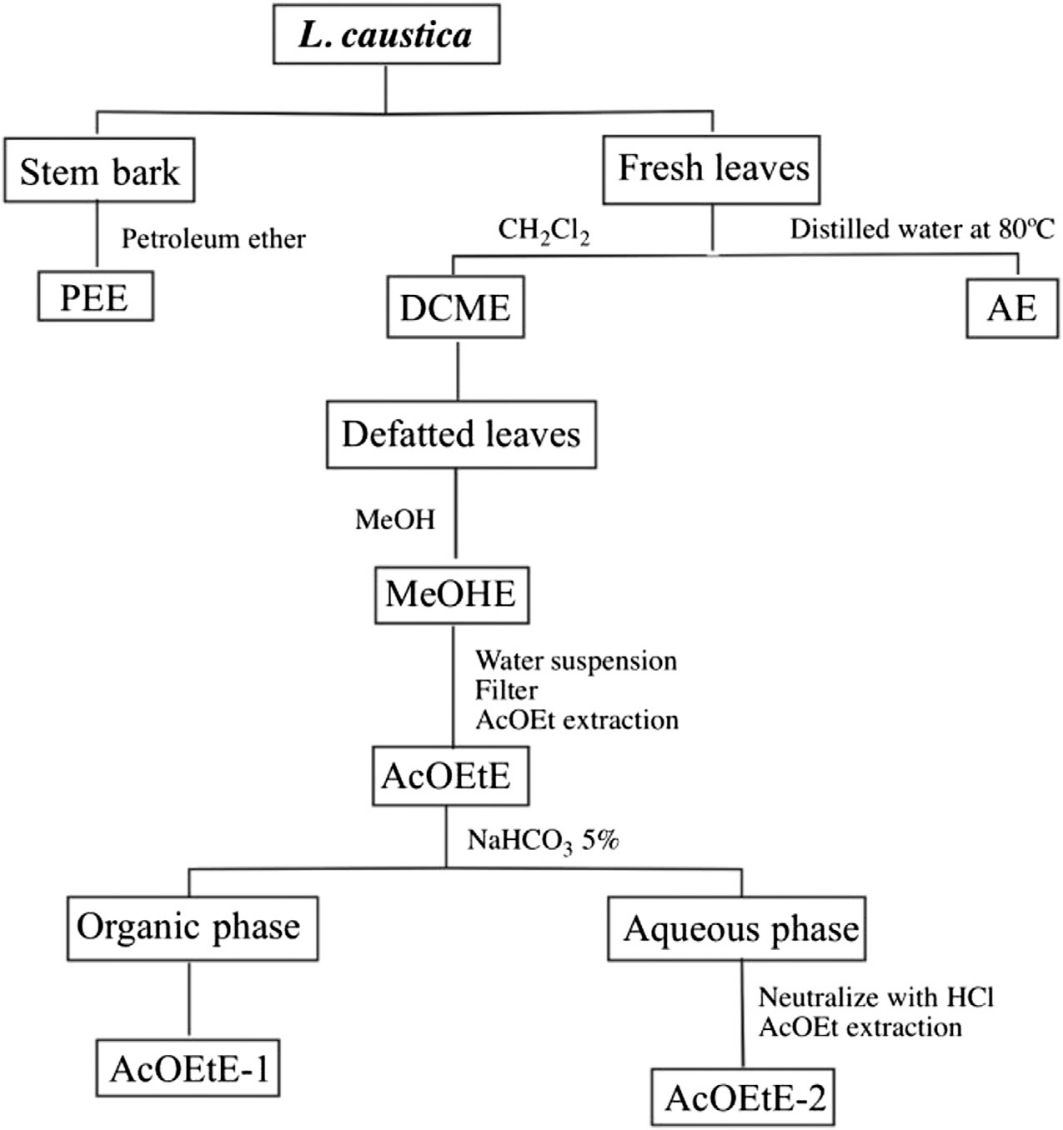
Extraction methodology. DCME, Dichloromethane extract from leaves; MeOHE, Methanol extract from defatted leaves; ACOEtE, Ethyl acetate fraction from the MeOHE; AcOEtE-1 and AcOEtE-2, sub-fractions from the ACOEtE; PEE, petroleum ether extract from stem bark; AE, aqueous extract.

Dried and milled stem bark of *L. caustica* (290 g) was extracted in Soxhlet apparatus for 8 h using 2.5 L of petroleum ether. The extract was dried over anhydrous sodium sulphate and filtered using a fritted glass funnel. The solvent was evaporated under reduced pressure in a rotatory evaporator, obtaining PEE (1.6 g, 0.55%) (Figure 1).

#### Dichloromethane Extract From Leaves

Leaves of *L. caustica* (1.4 kg), were extracted by dipping the fresh plant material in 5 L of cold CH_2_Cl_2_ for 5 min, for the extraction of the epicuticular components (Urzúa et al., 2011). The extract was dried over anhydrous sodium sulphate and filtered using a fritted glass funnel. The solvent was evaporated under reduced pressure in a rotatory evaporator obtaining DCME (6.1 g, 0.45% from fresh plant material) (Figure 1).

#### Ethyl Acetate Extract From Methanolic Extract

Dried defatted milled leaves of *L. caustica*, previously extracted with DCM (50 g), were extracted with 2 L MeOH using a Soxhlet apparatus for 4 h and the solvent was evaporated under reduced pressure in a rotatory evaporator, obtaining the MeOHE (14 g, 28% from plant material). The syrupy MeOHE was dissolved with 150 ml of H_2_O at 40°C, allowed to stand at room temperature and the suspension filtered using a fritted glass funnel. The solid was discarded and the filtrate extracted by liquid-liquid extraction with ethyl acetate (AcOEt) (4 × 30 ml). The solvent was evaporated under reduced pressure in a rotatory evaporator, obtaining AcOEtE (1.0 g, 2.0% from MeOHE) (Figure 1).

#### Fractionation of Ethyl Acetate Extract

The AcOEtE was re-suspended in 50 ml of AcOEt at 60°C and allowed to stand at room temperature. The suspension filtered using a fritted glass funnel. The solid was discarded and the filtrate extracted with 5% sodium bicarbonate (3 × 30 ml). The organic layer was dried over anhydrous sodium sulphate, filtered using a frit funnel and the solvent was evaporated under reduced pressure in a rotatory evaporator, obtaining AcOEtE-1 (140 mg, 14%, from AcOEtE; phenolic compounds fraction). The basic extract, kept at 0°C, was stirred and neutralized dropwise with concentrated hydrochloric acid, and extracted with AcOEt (4 × 30 ml). The AcOEt extract was dried over anhydrous sodium sulphate, filtered using a frit funnel and evaporated under reduced pressure in a rotatory evaporator, obtaining AcOEtE-2 (400 mg, 40%, from AcOEtE; acid-phenolic compounds fraction) (Figure 1). The AcOEtE-1 (phenolic compounds fraction) and AcOEtE-2 (acid-phenolic compounds fraction) were analyzed by LC-ESI-MS/MS.

#### Aqueous Extract From Leaves

Fresh leaves of *L. caustica* (314 g) were extracted to obtain an infusion, by dipping the fresh plant material in 700 ml of hot distilled water at 80°C for 5 min. The extract was filtered using a fritted glass funnel and the solvent was evaporated under reduced pressure in a rotatory evaporator obtaining AE (3.1 g, 0.98% from fresh plant material) (Figure 1).

#### Fractionation of Dichloromethane Extract and Petroleum Ether Extract: Catechols Isolation

Part of DCME (2.5 g) and PEE (1.6 g) were fractioned by column chromatography using silica gel and a solvent gradient of increasing polarity. For the DCME the elution gradient was as follows: light petroleum ether (PE) (bp 35–60°C), PE- CH_2_Cl_2_ (1:1), CH_2_Cl_2_, and CH_2_Cl_2_-MeOH (9:1). For the PE elution, gradient was as follows: light petroleum ether (PE) (bp 35–60°C), PE-CH_2_Cl_2_ (1:1) CH_2_Cl_2_, and CH_2_Cl_2_ step gradient. The fractions were evaporated under reduced pressure and were analyzed using thin layer chromatography on silica gel 60 F_254_ pre-coated plates, using 10% ferric chloride spray reagent for the detection of phenols. Fractions with similar chromatograms were combined and further purified by column chromatography, to produce 193 mg of a phenol fraction (catechols) from the DCME and 140 mg of a phenol fraction (catechols) from the PEE. Purity of the fractions and composition was obtained through thin-layer chromatography, GC-MS, FTIR and nuclear magnetic resonance (NMR) analysis.

#### Gas Chromatography-Mass Spectrometry Analysis

The analysis were performed in a gas chromatograph Shimadzu model GC-MS-QP 2010 Ultra (Shimadzu, Kyoto, Japan), operating in the splitless mode and fitted with a capillary GC column Rtx-5MS cross bond 5% diphenyl - 95% dimethyl polysiloxane (30 m length, 0.25 mm I.D., 0.25 µm film thickness) (Restek, Bellefonte, PA, United States). Analysis by GC-MS of the catechol fractions was done using the following conditions, column temperature was held at 40°C for 5 min, raised at 10 °C/min to 200°C and maintained for 5 min and the column temperature was raised at 3°C/min to 290°C and maintained for 20 min at 290°C. The injection volume was 1 µl and the carrier gas was helium (flow rate: 1.3 ml/min). The mass spectrometer was used in the electron impact ionization mode (70 eV) with an emission current of 250 µA and acquisition mass range, 50–500 Dalton. The temperatures of the injection port, ion source and transfer line were 250, 240 and 260°C, respectively. The instrument was operated in the scan mode. In the scan mode, the instrument monitors a wide and continuous range of masses determined by the molecular masses and fragmentation patterns of the potential compounds of interest. The identification of compounds in the chromatographic profiles were achieved by comparison of the compounds fragmentation with data from the literature.

#### Fourier Transform Infrared Spectroscopy Analysis

The samples were analyzed by Fourier transform infrared spectroscopy (FTIR), on a Bruker 66v Fourier-transform infrared spectroscopy spectrometer (4,000–400 cm^−1^). The samples were dissolved in methylene chloride and were analyzed in film.

#### High Performance Liquid Chromatography-Diode-Array-Detector Analysis

High performance liquid chromatography-diode-array-detector (HPLC-DAD) analysis were performed using liquid chromatograph (Waters 600; Milford, MA, United States) with a reverse-phase Symmetry Shield RP18 column (5-µm particle size; 25 × 0.46 cm). Gradient elution was performed using a mobile phase of 0.1% acetic acid in water (solution A) and 0.1% acetic acid in acetonitrile (solution B): 0–5 min, isocratic elution with 70% A/30% B; 5–45 min, linear gradient from 70 A/30 B to 55% A/45% B. A Waters 2996 DAD was used to detect the compounds and their spectra were recorded at wavelengths of 200–800 nm. Quantification was based on the areas of the peaks in the chromatograms, which were determined at 254 nm.

#### Nuclear Magnetic ResonanceAnalysis

Mono-dimensional ^1^H, ^13^C and DEPT-135, bi-dimensional homonuclear COSY, and heteronuclear bi-dimensional HSQC-ed and HMBC NMR spectrum were obtained on a Bruker DPX 400 spectrometer (400 MHz for ^1^H and 100 MHz for ^13^C). Samples were dissolved in CDCl_3_, and the spectra were calibrated using TMS signals. The chemical shifts are given in ppm.

#### Liquid Chromatography Electrospray Ionization Tandem Mass Spectrometric Analysis

The LC-ESI-MS/MS analysis were performed using a LC-ESI-MS/MS system consisted in a HPLC HP1100 (Agilent Technologies Inc., CA-United States) connected to the mass spectrometer Esquire 4000 Ion Trap LC/MS (n) system (Bruker Daltonik GmbH, Germany). A column Kromasil 100-5C18 of 250 × 4.6 mm, 5 μm and 100Å (Eka Chemicals AB, Sweden) was used for the analysis; at the exit of the column a split divided the eluent for simultaneous UV spectroscopy detection and mass spectrometry detection. The mobile phase was formic acid in water (0.1% v/v, solvent A) and formic acid in acetonitrile (0.1% v/v, solvent B) at a flow rate of 1 ml/min according to the following elution gradient: 0–5 min, 10% B; 5–20 min, 10–30% B; 20–52 min, 30–45% B; 52–53 min, 45–10% B and 53–60 min, 10% B. Compounds were detected at 254 nm. The mass spectral data were acquired in positive and negative modes; ionization was performed at 3,000 V assisted by nitrogen as nebulizing gas at 24 psi and as drying gas at 365°C and a flow rate of 6 L/min. All scans were performed in the range *m/z* 20–2,200. The trap parameters were set in ion charge control using manufacturer default parameters. Collision induced dissociation (CID) was performed by collisions with the helium background gas present in the trap and automatically controlled through Smart Defrag option. The tentative identification of the compounds in each fraction were based on: i) comparison of experimental fragmentation vs. library or literature fragmentation.; and ii) correlation between both polarities (however, some compounds were only observed in one ionization mode) and adduct presence. Those compounds observed in both polarities were labeled with M + H, M + Na or M−H.

### Derivatization Procedures

#### Hydrogenation of 3-n-alk(en)ylcatechols Mixture and 3-[(10Z)-Pentadec-10′-en-1-yl]-Catechol (2).

Samples of 3-*n*-alk(en)yl-catechols mixture of the phenolic fraction of *L. caustica* stem bark (25 mg) and 3-[(10*Z*)-pentadec-10′-en-1-yl]-catechol (**2**) (25 mg) and Pd/C (5 mg) in CH_2_Cl_2_ (2.5 ml) were respectively stirred under H_2_ at room temperature for 24 h. The reaction mixture was filtered, using a fritted glass funnel with Celite. The solvent was evaporated under reduced pressure in a rotatory evaporator, yielding the corresponding reduction products. The product of each reactions was purified by column chromatography and analyzed by GC-MS, FTIR and ^1^H and ^13^C NMR and identified in each reaction as 3-*n*-pentadecylcatechol (**3**) (Muñoz-Ramírez et al., 2020).

#### Acetylation of 3-[(10Z)-Pentadec-10′-en-1-yl]-Catechol (2) and 3-n-Pentadecylcatechol (3).

Independently, 3-[(10*Z*)-pentadec-10′-en-1-yl]-catechol (**2**) (22 mg) and of 3-*n*-pentadecylcatechol (**3**) (22 mg) were dissolved in 10 ml of dichloromethane and 0.7 ml of acetic anhydride followed of 40 mg by 4-*N*,*N*-dimethylaminopyridine (DMAP). The reaction mixture was stirred at room temperature for 24 h. The reaction mixture was successively washed with 5% solution of hydrochloric acid (2 ×x 3 ml), 6% solution of sodium bicarbonate (2 × 3 ml), and distilled water (2 × 3 ml). The resulting organic phase was dried over anhydrous sodium sulphate and filtered using a fritted glass funnel. The solvent was evaporated under reduced pressure in a rotatory evaporator, yielding the corresponding acetyl derivatives. The products from each reaction were purified by column chromatography and analyzed by FTIR and ^1^H and ^13^C NMR were identified as (*Z*)-1,2-diacetyl-3-(pentadec-10’-enyl)-benzene (**4**) and 1,2-diacetyl-3-pentadecylbenzene (**5**) (Muñoz-Ramírez et al., 2020).

### Evaluation of Bioactivity

#### *In Vitro* Assay of 15 Soybean Lipoxygenase Inhibition

Assays of inhibition of 15-sLOX were performed using a previous published methodology (Tirapegui et al., 2017), with minor modifications. In shortly, the activity of 15-sLOX (Cayman Chemical Item No. 60712) was determined following the formation of reaction products at 234 nm (ε = 25,000 M^−1^cm^−1^) with a Perkin-Elmer Lambda 25 UV/Vis (LabMakelaar Benelux B.V. Zuid-Holland, Nederland). All reactions were performed at a final volume of 2 ml and stirred using a magnetic bar at room temperature. The reaction medium used contained 0.1 M HEPES buffer (pH 7.4), Triton X-100 0.01%, and the linoleic acid substrate at a 10 μM concentration determine preliminary percent inhibition (% I). The concentration was determined quantitatively by allowing the enzymatic reaction to go to completion. The reaction was carried out by adding the inhibitor (sample) in methanol to the cuvette with the substrate buffer, and finally the enzyme was added. Nordihydroguaiaretic acid was used as positive control. Assays were performed in duplicate on two different days. IC_50_ values were determined by measuring enzyme activity at different concentrations of inhibitor dissolved in methanol; in the range of the initial velocity of the enzyme reaction. Finally, the inhibition% vs. the inhibitor concentration was plotted, giving a hyperbolic saturation curve using GraphPad Prism Demo.

#### *In Vitro* Assay of 5 Human Lipoxygenase Inhibition

Assays of inhibition of 5-hLOX were performed using a previous published methodology (Vásquez-Martínez et al., 2019), with minor modifications. In shortly, the enzyme (Cayman Chemical Item No. 60402) was diluted (1:500) in the assay buffer (HEPES 50 mM, EDTA 2 mM, ATP 10 μM and CaCl_2_ 10 μM at pH 7.5) and mixed with 10 μM of 2′,7′-dichlorofluorescin diacetate (H2DCFDA) dye. The reaction mixture was incubated for 15 min in the assay plate. Subsequently, 33.3 μl of assay buffer was added per well, and 3.3 μl of inhibitor was added to a final concentration of 10 μM; this reaction mixture was incubated for 30 min. The reaction was started by addition of AA (3.3 μM) and was incubated for 1 h and fluorescence was read in a multimode detector Synergy™ HT Multi-Mode Microplate Reader (Biotek, Winooski, VT, United States) at 480 nm excitation/ 520 nm emission after a reaction that had proceeded for 1 h at room temperature. The IC_50_ values were obtained using the nonlinear curve- fitting program of Graph Pad Prism Demo.

## Results and Discussion

### Analysis of the Catechols Fraction from the Petroleum Ether Extract From Stem Bark *Litharea Caustica* by Gas Chromatography-Mass Spectrometry

**FIGURE 2:**
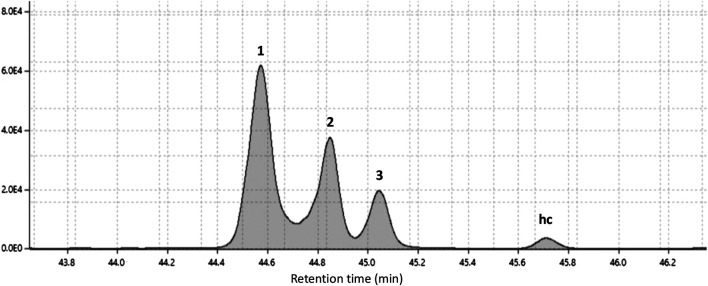
Gas chromatogram of catechols fraction from petroleum ether extract of stem bark of *Lithraea caustica.*

**FIGURE 3:**
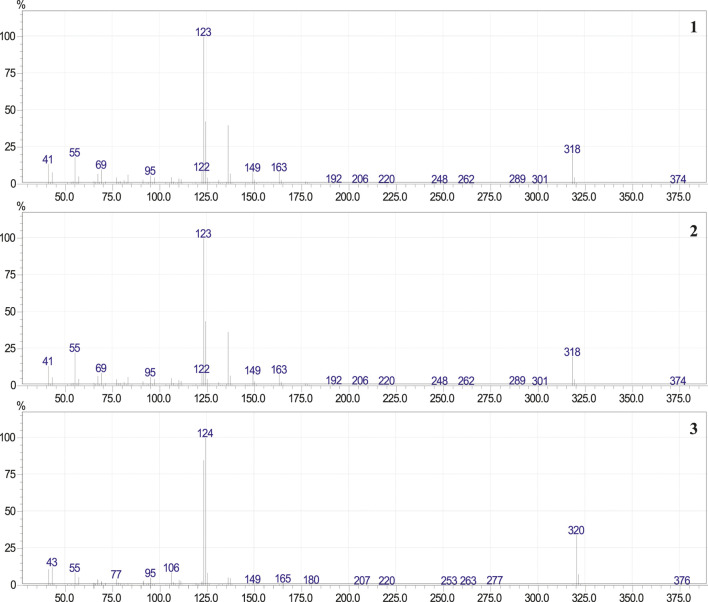
Mass spectra of 3-[(10*E*)-pentadec-10′-en-1-yl]-catechol (**1**), 3-[(10*Z*)-pentadec-10′-en-1-yl]-catechol (**2**) and 3-*n*-pentadecylcatechol (**3**).

The fraction of phenolic compounds from PEE, analyzed by GC-MS, correspond to a mixture of three compounds (Figure 2) and compounds **1** and **2** showed identical mass spectra, with molecular ion peaks at *m/z* 318, consistent with the molecular formula C_21_H_34_O_2_ (five unsaturations) and a base peak at *m/z* 123 (C_7_H_7_O_2_), consistent with a di-hydroxylated tropylium ion (Figure 3). The base peak of these spectra was coincident with the spectrum of 3-[(10*Z*)-pentadec-10′-en-1-yl]-catechol (**2**) previously isolated from *L. caustica* stem bark (Gambaro et al., 1986).

The mass spectra of compound (**3**) exhibit a molecular ion peak at *m/z* 320 consistent with the molecular formula C_21_H_36_O_2_ (four unsaturations) and two intense peaks at *m/z* 123 and 124 (C_7_H_7_O_2_ and C_7_H_8_O_2_, respectively), consistent with a di-hydroxylated tropylium ion. The main peaks of these spectra were coincident with previously reported spectrum for 3-*n*-pentadecycatechol (**3**) (Gross et al., 1975; Alé et al., 1997). To confirm the presence of compounds (**2**) and (**3**), the mixture 3-n-alk(en)yl catechols of the PEE, was analyzed by GC, using 3-[(10Z)-pentadec-10′-en-1-yl]-catecol (**2**) and 3-pentadecylcatecol (**3**) as standards. Observing the overlap of the chromatograms obtained for each sample analyzed (see Supplementary Figure S8 in the Supplementary Material).

### Characterization of 3-[(10Z)-Pentadec-10′-en-1-yl]-Catechol (2)

The catechols fraction from the DCME (193 mg), analyzed by GC-MS showed one intense peak with trace amounts of compound (**3**) (see Supplementary Figure S1 in Supplementary Material). Oil, UV *λ*_max_: 215.5, 253.1 and 323.0 nm; FTIR *ν*_max_, (film): 3441 cm^−1^ (O-H stretching), 3004.1 cm^-1^ (C_sp2_-H, stretching), 2953.15–2852.51 cm^−1^ (C-H, stretching) cm^−1^, EIMS: *m/z* 318 (M^+^) consistent with the molecular formula C_21_H_34_O_2_ (five unsaturations) and a base peak at *m/z* 123 consistent with the formula C_7_H_7_O_2_ (di-hydroxylated tropylium ion). The compound was directly subjected to NMR analysis. The ^13^C NMR and DEPT-135 spectrum of compound (**2**), confirms 21 carbon signals, corresponding to one methyl carbon, 12 methylene carbons, five sp^2^ methine carbons and three sp^2^ quaternary carbons. The ^1^H NMR spectrum showed one methyl group at *δ* 0.9 ppm (3H, *t*, *J* = 6.6), and twelve methylene groups at *δ* 1.26–1.40 (20H, m), *δ* 2.02 (4H, m) and *δ* 2.60 ppm (2H, *t*, *J* = 7.7), two olefinic protons at 5.36 ppm (2H, *t*, *J* = 4.6), and three aromatic protons at 6.71 ppm (3H, s). The methylene at *δ* 2.60 ppm (2H, *t*, *J* = 7.70) was assigned to one attached to a benzene ring and the two methylene at *δ* 2.02 ppm (4H, m) were attached to a double bond and coupled with the olefinic protons. Extensive 2D NMR experiments ^1^H–^1^H (COSY and NOESY) and ^1^H-^13^C (HMQC and HMBC) supported the structure of 3-[(10*Z*)-pentadec-10′-en-1-yl]-catechol (**2**) (see Figure 3; Supplementary Figure S2A–C in Supplementary Material), previously isolated from *L. caustica* stem bark (Gambaro et al., 1986). The (*Z*) double bond stereochemistry was obtained by correlation with *δ* values in the ^13^C NMR spectra of (Z) and (*E*) isomers of 9-tetradecene-1-yl acetate (Rossi, 1982).

### Characterization of 3-n-Pentadecylcatechol (3)

**FIGURE 4:**
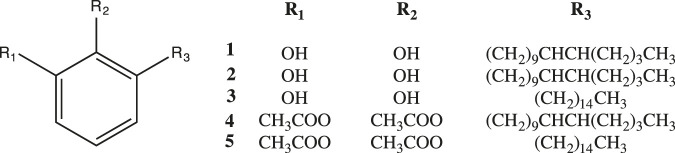
Structures of the isolated compounds [**1**-**3**] of *Litharea caustica* and its semisynthetic derivatives [**4** and **5**].

3-[(10*Z*)-pentadec-10′-en-1-yl]-catechol (**2**) (20 mg) and 3-*n*-alk(en)yl-catechols mixture (20 mg) of the phenolic fraction of *L. caustica* stem bark were hydrogenated yielding 3-*n*-pentadecycatechol (**3**). Solid, UV *λ*_max_: 194.4, 227.2 and 277.9 nm; FTIR *ν*_max_ (film): 3441 cm^−1^ (O-H stretching), 3004.1 cm^−1^ (C_sp2_-H, stretching), 2953.15–2852.51 cm^−1^ (C-H, stretching), EIMS: *m/z* 320 (M^+^) consistent with the molecular formula C_21_H_36_O_2_ (four insaturations) and one intense peak at *m/z* 123 (C_7_H_7_O_2_) identified as a di-hydroxylated tropylium ion. The compound was directly subjected to NMR analysis. The ^13^C NMR and DEPT-135 spectrums of compound **2**, confirms 21 carbon signals, corresponding to one methyl carbon, 14 methylene carbons, three sp^2^ methine carbons and three sp^2^ quaternary carbons. The ^1^H NMR spectrum showed one methyl group at δ 0.9 ppm (3H, *t*, *J* = 6.60 Hz), and thirteen methylene groups at: *δ* 1.26–1.40 ppm (26 H, m), and thee aromatic protons at 6.71 ppm (3H, s). The methylene at *δ* 2.60 ppm (2H, *t*, *J* = 7.70 Hz) was assigned to one attached to a benzene ring. Extensive 2D NMR experiments ^1^H–^1^H (COSY and NOESY) and ^1^H-^13^C (HMQC and HMBC) (see Supplementary Figure S3A–C in Supplementary Material) supported the structure of 3-*n*-pentadecyl-catechol (3) (Figure 4).

### Characterization of 3-[(10E)-Pentadec-10′-en-1-yl]-Catechol (1)

Compound (**1**) shows EIMS *m/z*: 318 (M^+^) consistent with the molecular formula C_21_H_34_O_2_ (five unsaturations) and a base peak at *m/z* 123 consistent with the formula C_7_H_7_O_2_ (di-hydroxylated tropylium ion). The 3-*n*-alk(en)yl-catechols mixture was subjected to NMR analysis. The ^13^C NMR spectra showed in the aromatic region, the same signals of compound (**3**) and (**2**) with only small differences in the chemical shifts. Two signals at *δ* 129.92 and 129.88 ppm, were assigned to C-10′ and C-11′; C-9′ and C-12′ at *δ* 29.8 and 30.0 [see Supplementary Figure S4A,B in Supplementary Material]. *E* stereochemistry of the double bond was obtained by correlation with δ values in the ^13^C NMR spectra of *Z* and *E* isomers of 9-tetradecene-1-yl-acetate (Rossi, 1982).

### Compounds Characterized in the Ethyl Acetate Extract-1 (Phenolic Compounds) and Ethyl Acetate Extract-2 (Acid-Phenolic Compounds) Fractions From Ethyl Acetate Extract

The fractions AcOEtE-1 and AcOEtE-2, were analyzed in positive and negative mode, by LC-ESI-MS/MS. Results are showed in Tables 1, 2. Table 1 contains the precursor *m/z* and fragmentations obtained in positive and negative polarity for the 28 chromatographic UV peaks detected for AcOEtE-2 (see Supplementary Figure S5 in Supplementary Material), the tentative identifications were based on: i) comparison of experimental fragmentation vs. library or literature fragmentation; and ii) correlation between both polarities (however, some compounds only were observed in one ionization mode) and adduct presence. Those compounds observed in both polarities were labeled with M + H, M + Na or M−H. The characterizations of some peaks, which appear repeated, are explained because correspond to isomers, as observed for: trigalloyl hexose (peaks 4, 5, 6, 7 and 10), myricetin-*O*-galloyl hexoside (peaks 7, 8, 9 and 15), myricetin-*O*-hexoside (peaks 10, 11 and 12), myricetin-*O*-rhamnoside (peaks 14, 15 and 17) and quercetin-*O*-galloyl hexoside (peaks 10, 13, 14, 20 and 25). Isomers are due to the different position of sugars or acid moiety such as for example in quercetin-*O*-galloyl hexosides with substitutions in the positions 3-2″, 3-6, 3-6″, 4′-6″ and 7-6″. Due to the complexity of extract, co-elution was observed for various peaks, this was elucidated with further examination of the fragmentation; as in the cases of peak 5 finally identified as trigalloyl hexose, peak 7 identified as HHDP-galloyl hexose and peak 20 assigned as quercetin-*O*-galloyl hexoside. Other compounds were identified by comparison with literature data as for example hydroxycinnamic acid-galloyl hexoside in peak 14, pentagalloyl hexose in peak 16, malic acid-digalloyl hexose in peak 21, myricetin-*O*-acetyl rhamnoside and myricetin-*O*-galloyl rhamnoside in peak 26. Peak 27 showed a *m/z* 505 signal with a fragmentation pattern different from that observed for signal *m/z* 505 from peak 23, a *m/z* 329 fragment probably due to loss of glucuronide residue, the fragments *m/z* 329, 316 and 301 probably formed by the successive loss of methyl groups in addition to the characteristic quercetin fragments *m/z* 151 and 179 suggesting based on the literature of the presence of quercetin-dimethyl ether-*O*-glucuronide (Falcão et al., 2013). The differentiation of luteolin or kaempferol derivatives was based on their characteristic fragments such as *m/z* 199 and 175 in the negative mode fragmentation of luteolin (Sánchez-Rabaneda et al., 2003; Sánchez-Rabaneda et al., 2004) or *m/z* 165 and 121 in the positive fragmentation of kaempferol (Cuyckens and Claeys, 2004; Justino et al., 2009). Other peaks were not characterized.

**TABLE 1:** Phytochemical compounds detected and characterized in *Lithraea caustica* leaves (AcOEtE-2, Phenolic-Acid Fraction), using liquid chromatography electrospray ionization tandem mass spectrometric in negative mode ionization.

PeakNo.	Retention time (min	[M-H]^-^(*m/z*)	Fragments ions (*m/z*)	Tentative assignment	Reference
1	3.1	326.9	168.4, 124.5	Gallic acid derivative	
2	4.3	169.2	124.2	Gallic acid	
5	11.6	635.2	483.0, 465.1, 301.1	Trigalloyl-hexose (isomer I)	Hooi Poay et al. (2011), Wyrepkowski et al. (2014)
6	13.3	289.6	244.7, 204.6, 178.7	*epi*-chatechin	
7	14.6	635.1	483.0, 465.1, 313.1	Trigalloyl-hexose (isomer II)	
		633.9		Hexahydroxydiphenic acid-galloyl-hexose	Gordon et al. (2011), Chisté and Mercadante (2012), Regueiro et al. (2014)
		631.7	479.0, 316.9	Myricetin-*O*-galloyl- hexoside (isomer I)	
8	15.3	454.1	326.9, 312.9, 168.4	Digalloyl-pentose	
9	15.7	632.1	479.2	Myricetin-*O*-galloyl-hexoside (isomer II)	
10	16.1	635.3	483.0, 465.0, 313.0	Trigalloyl-hexose (isomer III)	
		616.2	463.0, 301.0	Quercetin-*O*-galloyl-hexoside (isomer I)	
		480.0		Myricetin-*O*-hexoside (isomer I)	
12	16.8	480.4	316.4	Myricetin-*O*-hexoside (isomer II)	
13	17.1	615.7	463.1, 301.2	Quercetin-*O*-galloyl-hexoside (isomer II)	
14	17.6	615.5	463.0, 300.9	Quercetin-*O*-galloylhexoside (isomer III)	
		477.9	459.0, 433.2, 313.2, 300.8	Hydroxycinnamic acid-galloyl-hexoside	Zhao et al. (2013)
		463.1	316.1	Myricetin-*O*-rhamnoside (isomer I)	
15	18.0	631.1	479.0, 317.0	Myricetin-*O*-galloyl-hexoside (isomer III)	
16	18.4	938.7	787.1, 769.0	Pentagalloyl hexose	Wyrepkowski et al. (2014), Erşan et al. (2016)
17	18.7	477.2	300.8	Quercetin-*O*-glucuronide	
		463.4	316.0	Myricetin-*O*-rhamnoside (isomer II)	
18	19	463.3	300.8	Quercetin-*O*-hexoside	
20	19.6	615.5	493.1, 465.1, 461.6, 313.2, 301.2	Quercetin-*O*-galloyl hexoside (isomer IV)	Gordon et al. (2011), Erşan et al. (2016)
21	20.3	599.6	593.2, 479.1, 463.1, 313.2	Malic acid-digalloyl hexose	Abu-Reidah et al. (2015)
22	21.1	491.3	314.9	Isorhamnetin-*O*-glucuronide	
		477.6	314.1	Isorhamnetin-*O*-hexoside	
		447.3	300.8	Quercetin-*O*-rhamnoside	
23	21.5	505.5	463.0, 300.7	Quercetin-*O*-acetyl hexoside	
26	22.6	615.6	463.1, 300.9, 469.0, 317.0	Myricetin-*O*-galloyl rhamnoside	Saldanha et al. (2013), Abu-Reidah et al. (2015)
		506.2	462.9, 315.9, 300.9	Myricetin-*O*-acetyl rhamnoside	Gordon et al. (2011)
27	23.2	505.3	487.0, 444.9, 329.0, 316.1, 300.9	Quercetin-dimethylether-*O*-glucuronido	Falcão et al. (2013)
		431.4	284.7	Kaempferol-*O*-ramnoside	

**TABLE 2:** Phytochemical compounds detected and characterized in *Lithraea caustica* leaves (AcOEtE-1: Phenolic Fraction), using liquid chromatography electrospray ionization tandem mass spectrometric in negative mode ionization.

Peak No.	Retention time (min)	[M-H]^-^(*m/z*)	Fragments ions (*m/z*)	Tentative assignment	Reference
1	12.4	578.1	558.9, 451.0, 425.0, 407.0, 288.9	Procianidin dimer B	
2	13.3	635.0	483.0, 465.0	Trigalloyl-hexose	Mämmelä et al. (2000), Wyrepkowski et al. (2014)
		561.5	463.1, 301.7	*epi*-afzelechin-*epi*-catechin	
3	17.7	615.9	463.1, 301.7	Quercetin-*O*-galloylhexoside	
4	19.0	477.4	312.8, 312.8.1, 270.8, 168.4	Quercetin-*O*-glucuronide	
9	21.1	615.2	463.0, 300.9, 312.8	Quercetin-*O*-galloyl hexoside	Parejo et al. (2004), Erşan et al. (2016)
		447.3	300.7	Quercetin-*O*-rhamnoside	
13	22.6	505.8	463.0, 315.9, 300.8, 178.5	Quercetin-*O*-acetyl hexoside	
14	23.1	505.6	486.9, 444.9, 329.0 316.4, 300.9	Quercetin-dimethyl ether-*O*-glucuronide	
		461.6	314.0	Isorhamnetin-*O*-rhamnoside	
		431.5	284.7	Kaempferol-*O*-rhamnoside	
15	23.6	625.4	479.0, 316.9	Myricetin-*O*-rhamnosyl hexoside	Ding et al. (2008)
16	24.9	609.8	463.1, 300.9	Quercetin-*O*-rhamnosyl hexoside	
17	25.7	623.8	470.8, 314.8, 270.7	Isorhamnetin-*O*-galloyl (isomer I)	
18	26.2	624.2	470.8, 315.0, 299.9	Isorhamnetin-*O*-galloyl (isomer II)	
		594.1	284.8	Kaempferol-*O*-rhamnosyl hexoside (isomer I)	
19	29.0	547.4	462.9, 300.8	Quercetin-*O*-succinyl rhamnoside	
20	36.2	593.1	284.8	Kaempferol-*O*-rhamnosyl hexoside (isomer II)	
		537	443.0, 417.1, 399.2, 375.2	Biapigenin	Zhang et al. (2011), Yao et al. (2017)
21	37.0	315.4	299.8	Isorhamnetin	
		285.7		Kaempherol	
22	39.1	537.6	443.0, 417.3, 399.2, 375.3	Biapigenin	Zhang et al. (2011), Yao et al. (2017)
23	41.4	537.8	443.0, 417.1, 399.2, 375.3	Biapigenin	Zhang et al. (2011), Yao et al. (2017)

Table 2 contains the precursor *m/z* signals and fragmentations obtained in positive and negative polarity for the 25 chromatographic UV peaks detected for AcOEtE-1 extract (see Supplementary Figure S6 in Supplementary Material), the tentative identifications were based on the same parameters explained for the Table 1. Some identifications are repeated which is probably due to the presence of isomers as for trigalloyl hexose detected in peaks 1 and 2 and for the compounds quercetin-*O*-galloyl hexoside (peaks 3 and 9), quercetin-*O*-rhamnoside (peaks 7 and 9), kaempferol-*O*-rhamnosyl hexoside (peaks 18 and 20) where the isomers are mainly due to the different position of sugars moiety. Peaks 20, 22 and 23 showed the presence of biapigenin-type biflavones based on their fragmentation would correspond to amentoflavone, cuppressuflavone or other which differentiate by their apigenin inter-linkage. Co-elution was observed for various peaks which would be due to the complexity of extract. In some cases, the identification required a further examination of the fragmentation due to differences between experimental and library fragmentation as in the cases of peak 2 finally identified as trigalloyl hexose, of peaks 9 and 12 assigned as quercetin-*O*-galloyl hexoside. Other compounds were identified by comparison with literature data as for example quercetin-*O*-acetyl hexoside in peak 13, isorhamnetin-*O*-rhamnoside and quercetin-dimethyl ether-*O*-glucuronide in peak 14, myricetin-*O*-rhamnosyl hexoside in peak 15. For peak 4 was observed in positive polarity a signal *m/z* 601 with a fragment *m/z* 287 assigned as kaempferol, a fragment *m/z* 430 that would be due to the loss of gallic acid (171 Da), a low intensity fragment *m/z* 437 which would be due to the loss of an hexoside residue suggests the presence of a kaempferol galloyl derivative probably kaempferol-*O*-galloyl hexoside. Peak 13 showed in positive polarity the signal *m/z* 507 identified as quercetin-*O*-acetyl hexoside based on the similarity of its fragmentation with that of delphinidin-*O*-acetyl hexoside (Favretto and Flamini, 2000; Brito et al., 2014) however it showed the characteristic fragmentation of quercetin and confirmed the identification in negative polarity. Peaks 17 and 18 presented in negative polarity a signal *m/z* 624 that showed the same fragmentation pattern, a fragment *m/z* 315 identified as isorhamnetin, a fragment *m/z* 471 that would be formed by the loss of a gallic acid residue (152 Da), the data suggest the presence of an isorhamnetin galloyl derivative. Peak 19 was identified as quercetin-*O*-succinyl rhamnoside mainly based on the similarity of its fragmentation with that of peonidin-*O*-succinyl rhamnoside (De Brito et al*.*, 2007) but it showed the characteristic fragmentation of quercetin. The differentiation of luteolin or kaempferol derivatives was based on their characteristic fragments such as *m/z* 199 and 175 in the negative fragmentation of luteolin (Sánchez-Rabaneda et al*.*, 2003; Sánchez-Rabaneda et al*.*, 2004) or *m/z* 165 and 121 in the positive fragmentation of kaempferol (Cuyckens and Claeys, 2004; Justino et al., 2009). Other peaks were not characterized.

### *In Vitro* Assay of 15 Soybean Lipoxygenase and 5 Human Lipoxygenase Inhibition

The Table 3 shows the IC_50_ values against 15-sLOX and 5-hLOX. The extracts were evaluated on 15-sLOX and only AE was tested against 5-hLOX. The pure catechols and catechol mixture were tested against 15-sLOX and 5-hLOX. (see Supplementary Figure S7A–D in Supplementary Material).

**TABLE 3:** Inhibition of 15-sLOX and 5-hLOX from extracts and compounds from *Litharea caustica*.

Extracts and compounds	IC_50_ 15-sLOX Inhibition (µg/mL^a^ or µM^b^)	IC_50_ 5-hLOX Inhibition (µg/mL^a^ or µM^b^)
DCME	37.45^a^	NT
MeOHE	42.47^a^	
AcOEtE	70.69^a^	
AcOEtE-1	11.11^a^	
AcOEtE-2	24.14^a^	
AE	>250^a^	10.91^a^
Catechol mixture from PEE (included compound (**1**) 3-[(10E)-pentadec-10′-en-1-yl]-catechol in 65%.	11.77^a^ (37.0^b^)^*^	0.37^a^ (1.16^b^)^*^
3-[(10*Z*)-pentadec-10′-en-1-yl]-catechol (**2**)	54.77^b^	2.09^b^
3-pentadecylcatechol (**3**)	55.28^b^	2.74^b^
(*Z*)-1,2-diacetyl-3-(pentadec-10′-enyl)-benzene (**4**)	NA	
1,2-diacetyl-3-pentadecylbenzene (**5**)	NA	

5 human lipoxygenase; 5-hLOX, 15-sLOX. 15 soybean lipoxygenase; DCME, Dichloromethane extract from leaves; MeOHE, Methanol extract from leaves; ACOEtE, Ethyl acetate fraction from the MeOHE; AcOEtE-1 and AcOEtE-2: sub-fractions from the ACOEtE; PEE: petroleum ether extract from stem bark; AE: aqueous extract.

a Estimated considering MW 318. NT: Not tested, NA: Not active (>50 µg/mL). ^b^µM concentration.

Regarding the activity of the extracts of *L. caustica*, the DCME was the most potent inhibitor for 15-sLOX and the activity was correlated with the presence of 3-[(10*Z*)-pentadec-10′-en-1-yl]-catechol (**2**) in the leaves cuticle (Urzúa et al., 2011). The inhibition values (µg/ml) against 15-sLOX produced by extracts of *L. caustica* were comparable with active extracts from other plant species (Chung et al., 2009). In the sub-extracts, AcOEtE-1 and AcOEtE-2, obtained by fractionation of the AcOEtE (Figure 1.), phenols and phenol acids were respectively identified (Tables 1, 2). Several of the identified compounds have shown anti-inflammatory activity via LOX.

Among them are, gallic acid (Lee, 2019), epi-catechin (Schewe et al., 2002) and kaempferol (Deng et al., 2007). On the other hand, biapigenin (Jnawali et al., 2015) isorhamnetin (Manivannan and Shopna, 2015; Wang and Zhong, 2015), isorhamnetin-*O*-rhamnoside (Antunes-Ricardo et al., 2015), quercetin-*O*-galloylhexoside (Santos et al., 2017), and kaempferol (Deng et al., 2007) have shown anti-inflammatory activity by other routes of inhibition. 3-[(10Z)-pentadec-10′-en-1-yl]-catechol (**2**), 3-pentadecylcatechol (**3**) and the catechols mixture showed high inhibitory activity against 15-sLOX and 15-hLOX and were more selective against 15-hLOX. Compound (**2**), (**3**) and the catechols mixture showed IC_50_ values in the range of known flavonoid inhibitors of 5-hLOX: (−)-epicatechin IC_50_ = 22 μM; (−)-epigallocatechin gallate IC_50_ = 3.1 µM; quercetin IC_50_ = 0.6 µM (Sadik et al., 2003). Structural analysis of compounds (**1**), (**2**) and (**3**) and its relationship to 15-sLOX and 5-hLOX inhibitions showed structures with two functional groups, the catechol function and the presence of a double bond in compound (**2**) with Z stereochemistry and in the compound (**1**) (65%) with *E* stereochemistry. Acetylation of compound (**2**) and (**3**) completely eliminates the inhibitory activity, indicating that the catechol function is fundamental for enzymes inhibition. Finally, the infusion AE was evaluated against 15-sLOX and 5-hLOX. The extract showed no inhibition against 15-sLOX (IC_50_ > 250 µg/ml) considering it “Not active”; on the contrary, showed good inhibition against 5-hLOX (IC_50_ 10,91 µg/ml), validating *in vitro*, the anti-inflammatory use of *L. caustica* in Mapuche ethnomedicine.

## Conclusion

The epicuticular DCME showed high inhibitory activity against 15-sLOX and 5-hLOX and showed more selectivity against 5h-LOX, the activity was correlated with the presence of 3-[(10*Z*)-pentadec-10′-en-1-yl]-catechol (**2**) which also present inhibition of both enzymes isoforms. The AcOEtE, obtained from the MEOHE showed inhibition of 15-sLOX (IC_50_ 70.69 mg/L). The fractionation of AcOEtE showed two sub-fractions with greater activity. Analysis by HPLC-DAD and LC-ESI-MS/MS, confirmed a complex mixture of phenolic compounds in AcOEtE-1 and phenolic-acids in AcOEtE-2, several of them with known anti-inflammatory properties.

The catechol mixture obtained from the PEE also shows inhibition of both enzymes isoforms, being more active against 5-hLOX.

The inhibition values against 15-LOX and 5-LOX of 3-[(10Z) -pentadec-10′-en-1-yl]-catechol (**2**) and its saturated analog 3-pentadecylcatechol (3) (Table 3); showed that the presence of the double bond in the chain attached to the catechol is not an important structural feature for the inhibitory activity. In addition , the comparison of the inhibition values between 3-[(10Z)-pentadec-10′-en-1-yl]-catechol (**2**) and 3-[(10E)-pentadec-10′-en-1-yl]-catechol (**1**), [found in around 65% in mixture with (**2**) and (**3**)], clearly indicate that stereochemistry of the doble bond in the chain, is also not a fundamental structural feature for the activity.

The relevance of the previously detailed results, together with the fact that the AE of fresh leaves of *L. caustica*, showed good inhibitory activity against 5-hLOX, point in the direction of design new studies to validate *in vivo*, the traditional use of the decoction of *L. caustica* leaves and stems in Mapuche folk medicine, for treatment of joint inflammatory diseases and scaly skin lesions.

## Data Availability Statement

The raw data supporting the conclusions of this article will be made available by the authors, without undue reservation, to any qualified researcher.

## Author Contributions

AMR performed experiments, collected and analyzed data and contributed to the writing of the manuscript. CMC, AU and JE conceived the ideas. CMC contributed with the design and reagents for the biological assays. AB contributed with the LC-ESI-MS/MS analysis. AU and JE contributed to the writing of the manuscript and design of the research. All authors approved the final version of the manuscript.

## Conflict of Interest

The authors declare that the research was conducted in the absence of any commercial or financial relationships that could be construed as a potential conflict of interest.

## Abbreviations

15-sLOX, 15 soybean lipoxygenase; 5-hLOX, 5 human lipoxygenase; *L. caustica*, *Litharea caustica*; NDGA, nordihydroguaiaretic acid; LA, linoleic acid; AA, arachidonic acid; AE, aqueous extract; PEE, petroleum ether extract; MeOHE, methanolic extract; DCME, dichloromethane extract; AcOEtE, ethyl acetate extract; GC-MS, gas chromatography-mass spectrometry; NMR, Nuclear Magnetic Resonance; LC-ESI-MS/MS, Liquid Chromatography Electrospray Ionization Tandem Mass Spectrometric. TLC, Thin-layer chromatography; FT-IR, Fourier-transform infrared spectroscopy; HPLC-DAD, liquid chromatography with diode-array detector.
