# Supplementary material for: Inhibition of Soybean 15-Lipoxygenase and Human 5-Lipoxygenase by Extracts of Leaves, Stem Bark, Phenols and Catechols Isolated From Lithraea caustica (Anacardiaceae)
Source: Front Pharmacol. 2020 Nov 30;11:594257. doi: 10.3389/fphar.2020.594257 (PMC7774326; doi:10.3389/fphar.2020.594257)
Supplement: Supplementary file 1 [file datasheet1.docx]

**Supplementary material**

Figure S1. Chromatogram and mass spectra of phenolic fraction:
3-[(10*Z*)-pentadec-10-en-1-yl]-catechol





Figure S2(a). IR-FT spectra of the 3-[(10*Z*)-pentadec-10-en-1-yl]-catechol.

Figure S2(b). ^1^H NMR of 3-[(10*Z*)-pentadec-10-en-1-yl]-catechol.

Figure S2(c). ^13^C NMR of 3-[(10*Z*)-pentadec-10-en-1-yl]-catechol.

Figure S3(a). IR-FT spectra of 3-pentadecylcatechol.

Figure S3(b). ^1^H NMR of 3-pentadecylcatechol.

Figure S3(c). ^13^C NMR of 3-pentadecylcatechol.

Figure S4(a). ^1^H NMR of phenolic fraction of stem bark.

Figure S4(b). ^13^C NMR of phenolic fraction of stem bark.

Figure S5. UV peaks detected for AcOEtE-2.

Figure S6. UV peaks detected for AcOEtE-1 extract.

IC_50_: 42.47 µg/mL

IC_50_: 37.45 µg/mL

IC_50_: 70.69 µg/mL

IC_50_: 24.14 µg/mL

IC_50_: 11.11 µg/mL

Figure S7(a). IC_50_ graphics of DCME, MeOHE and AcOEtE as 15-sLOX inhibitors.

IC_50_: 10.91 µg/mL

Figure S7(b). IC_50_ graphic of Aqueous extract as 5-hLOX inhibitor.

IC_50_: 54.77 µM

IC_50_: 55.28 µM

Figure S7(c). IC_50_ graphics of 3-[(10*Z*)-pentadec-10-en-1-yl]-catechol and
3-pentadecylcatechol as 15-sLOX inhibitors.

1. (**B**)

IC_50_: 0.37 µg/mL

IC_50_: 11.77 µg/mL

Figure S7(d). IC_50_ graphics of phenolic fraction of stem bark as 15-sLOX (**A**) and 5-hLOX inhibitor (**B**).

**
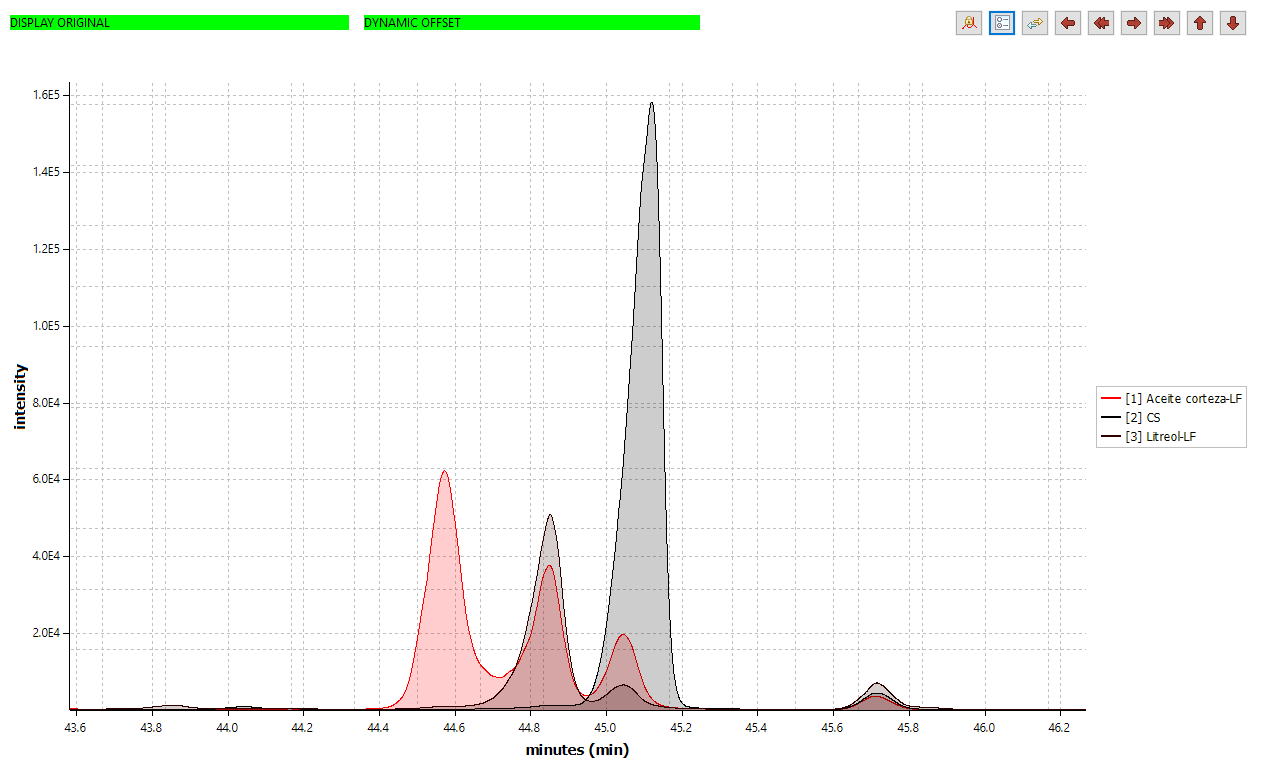
**

[1]

[2]

[3]

Catechol mixture

[1] 3-[(10*E*)-pentadec-10-en-1-yl]-catechol

[2] 3-[(10*Z*)-pentadec-10-en-1-yl]-catechol

[3] 3-pentadecylcatechol

Figure S8. GLC analysis of the catechol mixture from PEE was performed, using 3-[(10*Z*)-pentadec-10-en-1-yl]-catechol and 3-pentadecylcatechol as standards, superposition of the chromatograms.
